# Hyper-IgD syndrome/mevalonate kinase deficiency: what is new?

**DOI:** 10.1007/s00281-015-0492-6

**Published:** 2015-05-20

**Authors:** C. M. Mulders-Manders, A. Simon

**Affiliations:** Department of Internal Medicine, Nijmegen Centre for Immunodeficiency and Autoinflammation (NCIA), Radboud University Medical Center, Nijmegen, The Netherlands

## Abstract

Mevalonate kinase deficiency or hyper-IgD syndrome is a hereditary autoinflammatory syndrome caused by mutations in the mevalonate kinase gene. In this review, we will discuss new findings in this disorder that have been published in the last 2 years. This includes new insights into pathophysiology, treatment, and the clinical phenotype linked to the genetic defect.

## Introduction

The hyperimmunoglobulinemia D and periodic fever syndrome (HIDS) is an autoinflammatory disease characterized by recurrent episodes of fever, cervical lymphadenopathy, hepatomegaly, splenomegaly, abdominal pain, skin rash, arthralgia, and other inflammatory symptoms [1] accompanied by increased inflammatory markers such as C-reactive protein (CRP) and serum amyloid A (SAA). Febrile attacks can be triggered by childhood vaccinations or minor infection, although the triggers for most attacks are unknown. The name HIDS was derived from the fact that in the first case series, increased serum levels of immunoglobulin D (IgD) were found in all patients with this syndrome. Now that the genetic background is known, the currently more accurate name for the disorder is mevalonate kinase deficiency (MKD).

**Fig. 1:**
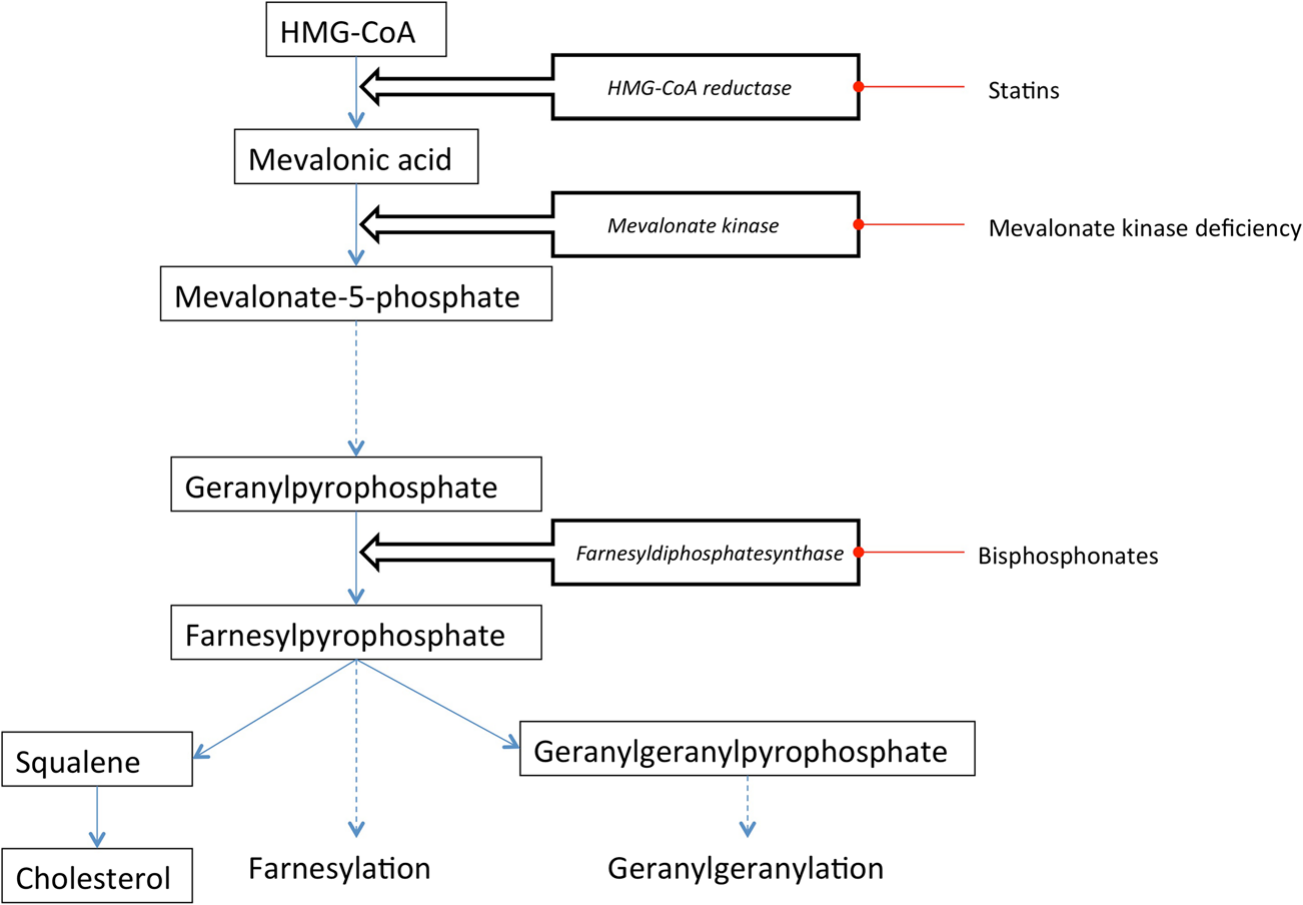
Mevalonate kinase catalyses the phosphorylation of mevalonic acid to 5-phosphomevalonate

MKD is caused by loss of function mutations in the mevalonate kinase gene (MVK) [2], which encodes for the protein mevalonate kinase. Mevalonate kinase is the second enzyme in the common pathway leading to both cholesterol and non-sterol isoprenoids and is located directly downstreams of HMG-CoA-reductase. Mevalonate kinase catalyses the phosphorylation of mevalonic acid to 5-phosphomevalonate (Fig. 1). Non-sterol isoprenoid end products are involved in the prenylation of proteins, where either a farnesyl group or a geranylgeranyl group is attached to a protein. This process is necessary for adequate protein function. Deficiency of mevalonate kinase leads to a shortage of intermediate compounds and end products of this pathway.

The level of remaining mevalonate kinase enzyme activity in MKD determines the clinical phenotype. Apart from the abovementioned clinical phenotype of HIDS, MKD can also present as mevalonate aciduria, a severe disease characterized by neurologic involvement with psychomotor retardation, cerebellar ataxia, and facial dysmorphy besides the inflammatory symptoms, leading to early death. MKD forms a continuous spectrum of disease between these two clinical entities. Overlapping clinical syndromes are seen with increasing frequency. As there is no clear border between phenotypes, we will use the term *mevalonate kinase deficiency*, which encompasses both HIDS and mevalonate aciduria, to describe the disease in this paper.

In this review, we will discuss new findings in MKD that have been published between January 1, 2012 and December 31, 2014.

### What is new on the pathophysiological mechanism of MKD?

In the past 30 years, MKD has been proven to be a typical monogenetic autoinflammatory disease with overproduction of the inflammatory cytokine interleukin-1 beta (IL-1β) as prominent pathophysiological mechanism [3–7]. The importance of this cytokine in MKD is backed up by the beneficial effects of IL-1β-targeting drugs such as anakinra in patients with this disease [8–11].

Most studies on the pathophysiology of MKD are based on in vitro cellular models with murine [12–14] or human cells with drug-induced block of the mevalonate kinase pathway with either HMG-CoA reductase inhibitors or bisphosphonates (Fig. 1). In these models, LPS or other bacterial components are used to mimic the inflammatory stimulus needed for the production of IL-1β. Stimulation of monocytes with LPS leads to increased pro-IL-1β transcription via activation of transcription factor NF-kB [5]. The effects of bisphosphonates on inflammation in mice were proven not to be strain-specific [15]. Besides cellular in vitro models, two in vivo animal models for MKD have been proposed. The first model uses heterozygote MVK knockdown mice that show a MKD-like phenotype with elevated body temperature, hepatosplenomegaly, and increased serum IgD [16]. With the use of this model, altered expression of stimulatory and inhibitory B7 glycoproteins on lymphocytes and macrophages with possible altered balance between stimulatory and inhibitory signals, as well as differences in cell proliferation between wild-type and knockdown mice, has been found. Interestingly, in these experiments, a difference between male and female mice was seen [17], although in humans, no gender-specific difference in clinical phenotype has been described.

In a second animal model of MKD, Balb/c mice are intraperitoneally injected with bisphosphonates 2 or 3 days prior to inflammatory stimulation followed by decapitation and analysis of inflammatory markers such as cytokines, peritoneal exudates, or splenic infiltration [15, 18, 19]. Comparison of the cytokine profile of these mice with the cytokine profile of healthy controls and MKD patients showed few discrepancies between the murine model and human monocytes, with increased IL-6 expression only in MKD patients and mouse-specific increased anti-TNF-α expression [20]. Extrapolation of the results from murine models to humans can be questioned as it has been proven that bisphosphonates might actually be able to be beneficial in MKD: A single patient with MKD treated with weekly bisphosphonates because of secondary osteoporosis showed complete resolution of the febrile episodes, which returned when the drug was ceased and disappeared again at reintroduction [21]. Thus, the pro-inflammatory effect of bisphosphonates may differ between species or between in vitro/in vivo experimental settings.

The link between increased IL-1β secretion and mevalonate kinase deficiency in MKD is most likely mediated by defective protein prenylation. In prenylation, non-sterol isoprenoids, such as farnesyl pyrophosphate (FPP) or geranylgeranyl pyrophosphate (GGPP) are coupled to a target protein, affecting its activity or cellular location. In a human monocytic MKD model, deficiency of GGPP leads to overproduction of IL-1β [22, 6, 23]. The deficiency of GGPP was shown to lead to defective prenylation of RhoA, rendering this protein inactive. Inactivity of RhoA results in increased activity of Rac1 and consequent activation of PKB [24]. Other small GTPases may also be affected. The Rac1/PI3K/PKB pathway had been linked to the pathogenesis of MKD earlier [5]. Liao et al. reported Rac1-independent increased IL-1β secretion in MKD [23]. Inactivation of RhoA was able to induce IL-1β mRNA transcription independent of NLRP3- or caspase-1 activity [24]. The B7 glycoproteins that were found to be affected in the earlier described murine MKD model are also prenylation-dependent [17].

Recently, several papers have focused on disrupted mitochondrial function in the pathophysiology of MKD. Possible involvement of mitochondria was suggested by the observation that in a murine cell model, inhibition of the mevalonate kinase pathway by HMG-CoA reductase inhibitors led to increased programmed cell death via caspase-3 and caspase-9, the latter being activated via an intrinsic mitochondrial pathway [25]. Mevalonate kinase deficiency leads to formation of elongated, instable mitochondria due to defective RhoA prenylation [14, 26, 24]. The aberrant mitochondria would normally be cleared from the cytosol by autophagia, but in MKD, clearance of the defective mitochondria is disrupted. Because of this defect, mitochondrial DNA accumulates in the cytosol and is able to bind and activate NLRP3, inducing IL-1β secretion [26]. Reactive oxygen species, but not specifically mitochondrial reactive oxygen species (mROS), are involved in this [26, 14]. Thus, both NLRP3-dependent as well as NLRP3-independent routes of IL-1β activation may be involved in the pathogenesis of MKD [26, 24].

IL-1β is not the only cytokine involved in the inflammatory response in MKD. PBMCs of MKD patients show a significantly different pattern of pro-inflammatory cytokine secretion after specific stimulation of several toll-like receptors (TLRs), including TLR4, TLR2, and nucleotide oligomerization domain-containing 2 (NOD2). Apart from IL-1β, there was increased expression of IL-1α, TNF, and IL-6 compared to PBMCs of healthy controls. Incubation of PBMCs from MKD patients with anakinra led to a normalization of cytokine expression (including TNF and IL-6) [11]. Marcuzzi et al. also observed increased expression of several different proinflammatory cytokines in their murine model [20]. These findings fit with the clinical observation that anti-IL-1β-treatment in MKD is not fully effective in all patients [11].

A probable explanation for the neurologic symptoms of severe MKD was suggested by Marcuzzi et al. In a neuroblastoma cell line, lovastatin was used as inhibitor of the mevalonate kinase pathway. Incubation of neuroblastoma cells with lovastatin led to a significant increase in programmed cell death with increased caspase-3 and caspase-9 activity, and this was inhibited by the addition of the isoprenoid end product GGOH [25]. Also, in mice, bisphosphonate treatment leads to microglial activation and intracerebral NLRP3 expression [15].

The enzyme deficiency in MKD results in an increased concentration of mevalonic acid, the substrate for mevalonate kinase, in plasma and urine. In mildly affected patients, this increase can be subtle, and accurate measurement can be tricky. Rodrigues et al. developed and validated an improved LC-MS/MS-based quantification assay for mevalonic acid which was tested in human as well as in rat samples [48].

### What is new on the treatment of mevalonate kinase deficiency?

The beneficial role of IL-1 targeting drugs as a therapy for MKD has been clear since the introduction of the IL-1 receptor antagonist anakinra. Anakinra binds the IL-1 receptor, preventing the actions of both IL-1α and IL-1β, and it has been shown to reduce the clinical and biochemical inflammation in MKD. It effectively decreases the frequency and severity of inflammatory attacks when used on a daily basis [8–10]. In patients with infrequent attacks, on-demand treatment (i.e., anakinra is started at the first signs of an inflammatory attack and ceased again after a few days) has been shown to be effective [8]. The major disadvantage of anakinra is the occurrence of painful injection site reactions. A single case report described clinical deterioration instead of improvement in a 12-year-old MKD patient when anakinra was initiated 2 days into a febrile attack with resolution of symptoms and inflammatory markers when anakinra was stopped again 5 days after it was started [27]. A possible concomitant infection aggravated by the IL-1-receptor blockage might explain these findings.

Canakinumab, a long acting monoclonal antibody directed against IL-1β has shown to be effective in reducing both frequency and severity in patients with mild and severe MKD in case reports and observational case series. Galeotti et al. describe six patients with MKD who were treated with canakinumab 2–7 mg/kg every 4 to 8 weeks. Three patients showed partial response with decreased frequency and severity of the inflammatory attacks. The other half had complete response, defined as no fever attacks and no inflammatory syndrome, during 10 to 21 months of treatment [28], and canakinumab was more effective than anakinra with regard to inhibiting the inflammatory response with fewer side effects in these patients [28]. Another patient who received canakinumab 4 mg/kg every 4 weeks as primary treatment for suspected MKD showed complete clinical response during 12 months of treatment, although biochemical inflammatory parameters remained elevated all this time [29]. In the Eurofever registry, a large international retrospective database on autoinflammatory diseases including 67 patients with MKD, complete response (no disease signs or symptoms and normalization of inflammatory markers) on canakinumab was seen in one of two patients, while the other one responded partially [10].

Most MKD patients benefit from anti-IL-1 therapy. However, anti-IL-1-resistant disease may occur. In a small number of case reports of patients unresponsive to anakinra, the effect of tocilizumab, a humanized monoclonal antibody against the interleukin-6 (IL-6) receptor, has been detailed. Shendi et al. treated a young woman in whom anakinra was ineffective with tocilizumab 8 mg/kg every 4 weeks (the standard dose scheme in rheumatoid arthritis) and found effective reduction of clinical and biochemical inflammation [30]. Two additional MKD patients treated with tocilizumab are described by Stoffels et al., who also observed reduction of frequency and severity of the inflammatory attacks, although after several months of treatment one of these two patients persistently showed mild inflammatory symptoms in the absence of biochemical inflammatory markers [11].

Anti-TNF therapy might be effective in MKD, but the effect is mostly partial and therapy failure and clinical deterioration have been described frequently in patients on infliximab or etanercept [10]. A beneficial effect of human monoclonal anti-TNFα antibody adalimumab was seen in a small number of MKD patients, where the two patients in the Eurofever registry that were treated with adalimumab showed only partial or no response [10, 31].

Several case reports, including one in the past 2 years, describe a beneficial effect of hematopoietic stem cell transplantation on the neurologic and inflammatory symptoms in severe mevalonate kinase deficiency [32–34]. Improvement of cerebral myelinisation on MRI after allogenic stem cell transplantation was seen in one girl [32]. This patient also showed resolution of spastic diplegia following liver transplantion, although this finding might be influenced by the start of physiotherapy directly after the transplantation. Liver transplantation did not influence febrile attacks in this patient [32]. These results could indicate that stem cell transplantation offers a curative option for severe MKD, especially for its neurological features.

### Extending the spectrum of mevalonate kinase deficiency

As previously described, MKD forms a spectrum of disease and can therefore present with a diversity of symptoms. In the past 2 years, it became clear that three more or less seemingly unrelated clinical presentations are associated with mutations in MVK (Table 1).

**Table 1:** Clinical presentations associated with mutations in the MVK gene

Typical autoinflammatory diseases	Mevalonic aciduria
	Hyperimmunoglobulinemia D and periodic fever syndrome (HIDS)
Inflammatory phenomena associated with MKD^a^	Ulcerative colitis
	Neonatal hepatitis
Diseases without systemic inflammation	Retinitis pigmentosa^b^
	Disseminated superficial actinic porokeratosis (DSAP)
	Porokeratosis of Mibelli

^a^Can be part of severe MKD phenotype and may be the initial presenting symptom in the absence of fever episodes

^b^Also, rare symptom of typical MKD

A new initial presentation of MKD was described in two patients with neonatal onset severe ulcerative colitis. In these patients, compound heterozygous mutations in MVK and increased urinary mevalonic acid excretion were found. Both patients presented with bloody diarrhea and abdominal pain. One of these patients also suffered from early onset fever; in the other, the first febrile episode occurred several months after the initial abdominal symptoms. Treatment with anakinra resulted in clinical and biochemical improvement in these patients [35]. A genome-wide association study in six children with mild to severe early onset colitis and a proven MVK mutation showed variants that have previously been linked to IBD in all of them [36], illustrating a possible genetic relation between MKD and IBD. IBD-like symptoms can indeed be part of the clinical phenotype of the HIDS-type of MKD as well.

Other rare or new presentations of MKD that were recently reported are cyclic neutropenia between febrile attacks in a young Israelian boy with homozygous V337I mutation [37], hepatitis [32, 38, 39], and macrophage activation syndrome in an American girl with compound heterozygous MVK mutations and a perforin polymorphism [38].

Retinitis pigmentosa is a clinical syndrome characterized by night blindness and peripheral vision loss. Retinitis pigmentosa has been described previously as a rare and severe complication of MKD. In a large cohort of retinitis pigmentosa patients lacking a genetic defect, three patients harboring homozygous mutations in MVK were found. These three patients seemed to have isolated retinitis pigmentosa, without a clinical phenotype of MKD otherwise, although two of these patients reported recurrent febrile episodes during childhood and had current mild symptoms that could be MKD related. The third patient did not have any signs of MKD. However, this patient was using a HMG-CoA reductase inhibitor because of ischemic heart disease, which may have influenced the phenotype. Severely decreased serum mevalonate kinase activity, and increased urinary mevalonic acid excretion was found in all three patients. Two out of three showed increased serum IgD concentrations [40].

Disseminated superficial actinic porokeratosis (DSAP) is an autosomal dominant hereditary skin disease characterized by annular keratotic lesion predominantly located on sun-exposed parts of the skin. Heterozygous mutations in MVK were identified by exome sequencing in several familial and sporadic DSAP cases [41–43]. Several more case reports have since appeared [44–46], all of them from patients of Asiatic origin. Although some of the mutations identified have also been found in MKD, most mutations were splice site mutations, the studied DSAP patients did not show any clinical signs of MKD, and porokeratosis is not a known feature of MKD [41, 44]. In DSAP, no abnormal IgD serum levels have been described [41]. The effect of the MVK mutations associated with DSAP on enzyme activity has not been studied to date. In a Chinese family with porokeratosis of Mibelli, a dermatological disease closely related to DSAP, a novel splice site mutation in MVK was found [47]. A possible causative link between MVK mutations and porokeratosis is that mevalonate kinase prevents UVA-induced apoptosis in keratinocytes [41].

## Conclusion

In the last 2 years, research on MKD has covered a range of subjects in pathogenesis and therapy. Also, the already broad spectrum of MKD phenotypes has been extended to include retinitis pigmentosa, colitis, and DSAP.

## References

[1] van der Meer JW, Vossen JM, Radl J, van Nieuwkoop JA, Meyer CJ, Lobatto S, van Furth R (1984). Hyperimmunoglobulinaemia D and periodic fever: a new syndrome. Lancet.

[2] Drenth JP, Cuisset L, Grateau G, Vasseur C, van de Velde-Visser SD, de Jong JG, Beckmann JS, van der Meer JW, Delpech M (1999). Mutations in the gene encoding mevalonate kinase cause hyper-IgD and periodic fever syndrome. International Hyper-IgD Study Group. Nat Genet.

[3] Drenth JP, Goertz J, Daha MR, van der Meer JW (1996). Immunoglobulin D enhances the release of tumor necrosis factor-alpha, and interleukin-1 beta as well as interleukin-1 receptor antagonist from human mononuclear cells. Immunology.

[4] Frenkel J, Rijkers GT, Mandey SH, Buurman SW, Houten SM, Wanders RJ, Waterham HR, Kuis W (2002). Lack of isoprenoid products raises ex vivo interleukin-1beta secretion in hyperimmunoglobulinemia D and periodic fever syndrome. Arthritis Rheum.

[5] Kuijk LM, Beekman JM, Koster J, Waterham HR, Frenkel J, Coffer PJ (2008). HMG-CoA reductase inhibition induces IL-1beta release through Rac1/PI3K/PKB-dependent caspase-1 activation. Blood.

[6] Kuijk LM, Mandey SH, Schellens I, Waterham HR, Rijkers GT, Coffer PJ, Frenkel J (2008). Statin synergizes with LPS to induce IL-1beta release by THP-1 cells through activation of caspase-1. Mol Immunol.

[7] Pontillo A, Paoluzzi E, Crovella S (2010). The inhibition of mevalonate pathway induces upregulation of NALP3 expression: new insight in the pathogenesis of mevalonate kinase deficiency. Eur J Human Genet : EJHG.

[8] Bodar EJ, Kuijk LM, Drenth JP, van der Meer JW, Simon A, Frenkel J (2011). On-demand anakinra treatment is effective in mevalonate kinase deficiency. Ann Rheum Dis.

[9] Bodar EJ, van der Hilst JC, Drenth JP, van der Meer JW, Simon A (2005). Effect of etanercept and anakinra on inflammatory attacks in the hyper-IgD syndrome: introducing a vaccination provocation model. Neth J Med.

[10] Ter Haar N, Lachmann H, Ozen S, Woo P, Uziel Y, Modesto C, Kone-Paut I, Cantarini L, Insalaco A, Neven B, Hofer M, Rigante D, Al-Mayouf S, Touitou I, Gallizzi R, Papadopoulou-Alataki E, Martino S, Kuemmerle-Deschner J, Obici L, Iagaru N, Simon A, Nielsen S, Martini A, Ruperto N, Gattorno M, Frenkel J, Paediatric Rheumatology International Trials O, The Eurofever/Eurotraps Project (2013). Treatment of autoinflammatory diseases: results from the Eurofever Registry and a literature review. Ann Rheum Dis.

[11] Stoffels M, Jongekrijg J, Remijn T, Kok N, van der Meer JW, Simon A (2015). TLR2/TLR4-dependent exaggerated cytokine production in hyperimmunoglobulinaemia D and periodic fever syndrome. Rheumatology.

[12] Marcuzzi A, Tommasini A, Crovella S, Pontillo A (2010). Natural isoprenoids inhibit LPS-induced-production of cytokines and nitric oxide in aminobisphosphonate-treated monocytes. Int Immunopharmacol.

[13] Marcuzzi A, Piscianz E, Girardelli M, Crovella S, Pontillo A (2011). Defect in mevalonate pathway induces pyroptosis in Raw 264.7 murine monocytes. Apoptosis : Int J Programmed Cell Death.

[14] Tricarico PM, Kleiner G, Valencic E, Campisciano G, Girardelli M, Crovella S, Knowles A, Marcuzzi A (2014). Block of the mevalonate pathway triggers oxidative and inflammatory molecular mechanisms modulated by exogenous isoprenoid compounds. Int J Mol Sci.

[15] Kleiner G, Celsi F, Tricarico PM, Zacchigna S, Crovella S, Marcuzzi A (2013). Systemic and neuronal inflammatory markers in a mouse model of mevalonate kinase deficiency: a strain-comparative study. In Vivo.

[16] Hager EJ, Tse HM, Piganelli JD, Gupta M, Baetscher M, Tse TE, Pappu AS, Steiner RD, Hoffmann GF, Gibson KM (2007). Deletion of a single mevalonate kinase (Mvk) allele yields a murine model of hyper-IgD syndrome. J Inherit Metab Dis.

[17] Hager EJ, Piganelli JD, Tse HM, Gibson KM (2012). Aberrant expression of costimulatory molecules in splenocytes of the mevalonate kinase-deficient mouse model of human hyper-IgD syndrome (HIDS). J Inherit Metab Dis.

[18] Marcuzzi A, Pontillo A, De Leo L, Tommasini A, Decorti G, Not T, Ventura A (2008). Natural isoprenoids are able to reduce inflammation in a mouse model of mevalonate kinase deficiency. Pediatr Res.

[19] Marcuzzi A, Crovella S, Pontillo A (2011). Geraniol rescues inflammation in cellular and animal models of mevalonate kinase deficiency. In vivo.

[20] Marcuzzi A, Zanin V, Kleiner G, Monasta L, Crovella S (2013). Mouse model of mevalonate kinase deficiency: comparison of cytokine and chemokine profile with that of human patients. Pediatr Res.

[21] Cantarini L, Vitale A, Magnotti F, Lucherini OM, Caso F, Frediani B, Galeazzi M, Rigante D (2013). Weekly oral alendronate in mevalonate kinase deficiency. Orphanet J Rare Dis.

[22] Mandey SH, Kuijk LM, Frenkel J, Waterham HR (2006). A role for geranylgeranylation in interleukin-1beta secretion. Arthritis Rheum.

[23] Liao Y-H, Lin Y-C, Tsao S-T, Lin Y-C, Yang A-J, Huang C-T, Huang K-C, Lin WW (2013). HMG-CoA reductase inhibitors activate caspase-1 in human monocytes depending on ATP release and P2X7 activation. J Leukoc Biol.

[24] van der Burgh R, Pervolaraki K, Turkenburg M, Waterham HR, Frenkel J, Boes M (2014). Unprenylated RhoA contributes to IL-1beta hypersecretion in mevalonate kinase deficiency model through stimulation of Rac1 activity. J Biol Chem.

[25] Marcuzzi A, Zanin V, Piscianz E, Tricarico PM, Vuch J, Girardelli M, Monasta L, Bianco AM, Crovella S (2012). Lovastatin-induced apoptosis is modulated by geranylgeraniol in a neuroblastoma cell line. Int J Dev Neurosci : Off J Int Soc Developmental Neurosci.

[26] van der Burgh R, Nijhuis L, Pervolaraki K, Compeer EB, Jongeneel LH, van Gijn M, Coffer PJ, Murphy MP, Mastroberardino PG, Frenkel J, Boes M (2014). Defects in mitochondrial clearance predispose human monocytes to interleukin-1beta hypersecretion. J Biol Chem.

[27] Shendi HM, Walsh D, Edgar JD (2012). Etanercept and anakinra can prolong febrile episodes in patients with hyperimmunoglobulin D and periodic fever syndrome. Rheumatol Int.

[28] Galeotti C, Meinzer U, Quartier P, Rossi-Semerano L, Bader-Meunier B, Pillet P, Kone-Paut I (2012). Efficacy of interleukin-1-targeting drugs in mevalonate kinase deficiency. Rheumatology.

[29] Tsitsami E, Papadopoulou C, Speletas M (2013). A case of hyperimmunoglobulinemia d syndrome successfully treated with canakinumab. Case Rep Rheumatol.

[30] Shendi HM, Devlin LA, Edgar JD (2014). Interleukin 6 blockade for hyperimmunoglobulin D and periodic fever syndrome. J Clin Rheumatol : Pract Rep Rheum Musculoskelet Dis.

[31] Di Gangi M, Amato G, Converso G, Benenati A, Leonetti C, Borella E, Doria A, Foti R (2014). Long-term efficacy of adalimumab in hyperimmunoglobulin D and periodic fever syndrome. Isr Med Assoc J.

[32] Chaudhury S, Hormaza L, Mohammad S, Lokar J, Ekong U, Alonso EM, Wainwright MS, Kletzel M, Whitington PF (2012). Liver transplantation followed by allogeneic hematopoietic stem cell transplantation for atypical mevalonic aciduria. Am J Transplant : Off J Am Soc Transplant Am Soc Transplant Surg.

[33] Neven B, Valayannopoulos V, Quartier P, Blanche S, Prieur AM, Debre M, Rolland MO, Rabier D, Cuisset L, Cavazzana-Calvo M, de Lonlay P, Fischer A (2007). Allogeneic bone marrow transplantation in mevalonic aciduria. N Engl J Med.

[34] Arkwright PD, Abinun M, Cant AJ (2007). Mevalonic aciduria cured by bone marrow transplantation. N Engl J Med.

[35] Levy M, Arion A, Berrebi D, Cuisset L, Jeanne-Pasquier C, Bader-Meunier B, Jung C (2013). Severe early-onset colitis revealing mevalonate kinase deficiency. Pediatrics.

[36] Bianco AM, Girardelli M, Vozzi D, Crovella S, Kleiner G, Marcuzzi A (2014). Mevalonate kinase deficiency and IBD: shared genetic background. Gut.

[37] Parvaneh N, Ziaee V, Moradinejad MH, Touitou I (2014). Intermittent neutropenia as an early feature of mild mevalonate kinase deficiency. J Clin Immunol.

[38] Schulert GS, Bove K, McMasters R, Campbell K, Leslie N, Grom AA (2014). Mevalonate kinase deficiency associated with recurrent liver dysfunction, macrophage activation syndrome and perforin gene polymorphism. Arthritis Care Res.

[39] von Linstow M-L, Rosenfeldt V (2014). Neonatal hepatitis as first manifestation of hyperimmunoglobulinemia d syndrome. Case Rep Pediatr.

[40] Siemiatkowska AM, van den Born LI, van Hagen PM, Stoffels M, Neveling K, Henkes A, Kipping-Geertsema M, Hoefsloot LH, Hoyng CB, Simon A, den Hollander AI, Cremers FPM, Collin RWJ (2013). Mutations in the mevalonate kinase (MVK) gene cause nonsyndromic retinitis pigmentosa. Ophthalmology.

[41] Zhang S-Q, Jiang T, Li M, Zhang X, Ren Y-Q, Wei S-C, Sun L-D, Cheng H, Li Y, Yin X-Y, Hu Z-M, Wang Z-Y, Liu Y, Guo B-R, Tang H-Y, Tang X-F, Ding Y-T, Wang J-B, Li P, Wu B-Y, Wang W, Yuan X-F, Hou J-S, Ha W-W, Wang W-J, Zhai Y-J, Wang J, Qian F-F, Zhou F-S, Chen G, Zuo X-B, Zheng X-D, Sheng Y-J, Gao J-P, Liang B, Li P, Zhu J, Xiao F-L, Wang P-G, Cui Y, Li H, Liu S-X, Gao M, Fan X, Shen S-K, Zeng M, Sun G-Q, Xu Y, Hu J-C, He T-T, Li Y-R, Yang H-M, Wang J, Yu Z-Y, Zhang H-F, Hu X, Yang K, Wang J, Zhao S-X, Zhou Y-W, Liu J-J, Du W-D, Zhang L, Xia K, Yang S, Wang J, Zhang X-J (2012). Exome sequencing identifies MVK mutations in disseminated superficial actinic porokeratosis. Nat Genet.

[42] Dai J, Chen M, Fu X, Yu Y, Shi Z, Yu C, Wang G, Tian H, Liu H, Zhang F (2013). Mutation analysis of the MVK gene in Chinese patients with disseminated superficial actinic porokeratosis. J Dermatol Sci.

[43] Zhou Y, Liu J, Fu X, Yu Y, Shi B, Yu G, Shi Z, Wu W, Pan F, Tian H, Liu H, Zhang F (2013). Identification of three novel frameshift mutations of the MVK gene in four Chinese families with disseminated superficial actinic porokeratosis. Br J Dermatol.

[44] Lu WS, Zheng XD, Yao XH, Zhang LF, Wang MQ, Jiang FX, Zhang SP, Hu B (2014). A novel MVK missense mutation in one Chinese family with disseminated superficial actinic porokeratosis. Mol Biol Rep.

[45] Zhou MS, Xie HF, Chen ML, Jian D, Liu FF, Chen X, Shen N, Si N, Li J (2014). A novel mutation for disseminated superficial actinic porokeratosis in the MVK gene. Br J Dermatol.

[46] Lu WS, Zheng XD, Yao XH, Zhang LF, Hu B, Lu YJ (2014). Detection of a novel missense mutation in the mevalonate kinase gene in one Chinese family with DSAP. Int J Clin Exp Pathol.

[47] Zeng K, Zhang QG, Li L, Duan Y, Liang YH (2014). Splicing mutation in MVK is a cause of porokeratosis of Mibelli. Arch Dermatol Res.

[48] Rodrigues AVM, Maggs JL, McWilliam SJ, Pirmohamed M, Coen M, Wilson ID, Park BK, Antoine DJ (2014). Quantification of urinary mevalonic acid as a biomarker of HMG-CoA reductase activity by a novel translational LC-MS/MS method. Bioanalysis.

